# The Effect of Aging on the Microstructure and Mechanical Properties of Solidified Lead-Bismuth Eutectic Alloy

**DOI:** 10.3390/ma18092001

**Published:** 2025-04-28

**Authors:** Hailuo Zhong, Sijuan Chen, Weibing Liao, Jiawei Zhang, Xuan Xiao, Xi Huang

**Affiliations:** College of Physics and Optoelectronic Engineering, Shenzhen University, Shenzhen 518060, China; 2017183017@email.szu.edu.cn (H.Z.); chensijuan@szu.edu.cn (S.C.); liaowb@szu.edu.cn (W.L.);

**Keywords:** lead-bismuth eutectic alloy, aging, microstructure, volume expansion mechanism, mechanical properties

## Abstract

Lead-bismuth eutectic (LBE) is a eutectic alloy of lead (44.5 at%) and bismuth (55.5 at%) that can be used as the coolant for the fast nuclear reactors. In the event of specific conditions or even accidents of the reactors, the temperature of liquid LBE decreases, and it may undergo solidification and volume expansion during the aging process after solidification, which can easily cause damage to the reactor’s internal structure as well as the reactor vessels. In this study, the microstructure and mechanical properties of solidified LBE obtained at different cooling rates are systematically investigated after different aging times. It was found that the internal structure of LBE after aging remained a eutectic microstructure, consisting of the γ-phase (Bi-rich phase) and β-phase (Pb_7_Bi_3_). After a long period of static aging, the white γ-phase precipitated into the black β-phase, which further confirms the phase transition mechanism. Meanwhile, the acceleration of the cooling rate can aggravate volume expansion. As the aging time increases, there is no significant difference in the compressive yield strength σ of the LBE samples with the same cooling rate and only a certain degree of fluctuation. The elastic modulus E also shows similar results, indicating that aging time has a minor effect on the compressive yield strength σ and elastic modulus E of the LBE. With the increase in cooling rate, the compressive yield strength σ shows an upward trend, while the elastic modulus E is not significantly affected, with a small amplitude of fluctuation. Meanwhile, the hardness of LBE samples after long-term aging treatment is enhanced. After long-term aging, the overall density of the LBE samples shows a decreasing trend, the density fluctuation range of the fast cooling rates (5 K/min and 10 K/min) are significantly larger than that of the slow cooling rates. The decrease in density leads to volume expansion of the LBE during the aging process after solidification.

## 1. Introduction

Lead-bismuth eutectic (LBE) is one of the candidate coolants for the proposed Generation IV nuclear reactors due to its advantageous properties. Its relatively low melting point (Tm = 123.5 °C) mitigates the risk of LBE freezing during operation, while its high boiling point and minimal reactivity with oxygen and nitrogen significantly reduce the reactor operational risks and improve the thermal efficiency.

However, a notable phenomenon is that LBE in the primary loop of nuclear reactors can undergo solidification as a result of a temperature decrease and then volume expansion after solidification, which may occur in the event of specific reactor operational incidents or accidents. This will potentially exert substantial forces on the internal structures of nuclear reactors as well as the main vessels, pipelines, or coolant storage tanks [1,2,3,4,5]. Typically, LBE undergoes volume contraction during the very first stages of solid-phase formation, subsequently exhibiting gradual expansion during the solidification and aging processes [6], even if the temperature does not change afterwards. Relevant studies have analyzed the density change, yield strength, and volume expansion mechanism of LBE post-solidification [1,2,3,4,5,6,7,8,9,10,11,12,13,14,15,16,17,18,19,20,21]. Glasbrenner et al. investigated the results of the temporal expansion of LBE following rapid cooling and solidification [12]. During the process of solidification, the LBE samples experienced a volume contraction of approximately 0.35%. This volume change was reversed, and the samples started to expand in approximately 100 min while the samples were maintained constant at room temperature. After approximately 10^5^ min, the rate of volume expansion of LBE began to decrease. In about 1 year, the volume increased by about 1.2%. During this period, the samples of LBE exhibited significant microstructure changes. The phenomenon of LBE expansion persisting for over six months without reaching equilibrium was also observed in the research conducted by Takeda [7]. In the study, following the solidification, the metallographic organization of the LBE entered an aging phase. During this period, the metallographic organization exhibited dynamic behavior, undergoing complex transformations. These transformations not only affected the internal structure of LBE but also significantly affected the evolution of its mechanical properties. Specifically, the key physical properties of LBE, including the density, yield strength, and elastic modulus, underwent changes during the aging process. The post-solidification LBE exhibited a eutectic fishbone microstructure, comprising a γ-phase (Bi-rich phase) and a β-phase (Pb_7_Bi_3_). Furthermore, LBE samples subjected to rapid cooling during solidification exhibited more fine-grained microstructures during the room temperature aging process [13]. As the volume of LBE expands during aging, complex interactions between LBE and the container wall or internal structures occur. The potential deformation and degradation of the mechanical performance of the structures can not only be brought about by the expansion or density change of LBE but can also depend on other mechanical properties of LBE, particularly the yield strength.

These studies have qualitatively investigated volume expansion during the aging process of LBE after solidification. The basic characteristics and trends of volume expansion have been revealed to a certain extent. However, quantitative studies provide more data, and detailed analyses are scarcely reported. This study aims to systematically study microstructure evolution and changes in mechanical properties over time during the aging process of solidified LBE obtained by means of different sample preparation, i.e., different cooling rates during solidification.

A series of experiments was set up and implemented. The microstructure, density, and mechanical properties of solidified LBE samples under various experimental conditions were analyzed, aiming to comprehensively observe the microstructure evolution of LBE during the aging process, including changes in the proportion of different phases and morphology characteristics. Meanwhile, the changes in physical properties, including the yield strength σ and the elastic modulus E of LBE over time, were systematically measured and reported. Through these comprehensive experiments and analyses, the effects of aging on the microstructure and mechanical properties of LBE are revealed, providing a solid theoretical and experimental basis for the optimization of the structural design and material selection of LBE-cooled fast nuclear reactors.

## 2. Materials and Methods

The cooling rates for the sample preparation of LBE were selected as 10 K/min, 5 K/min, 1 K/min, 0.1 K/min, and the initial temperature of the preparation was 423 K, which is beyond the freezing point of 396.65 K. The cutoff temperature was set to 300 K. Cylindrical LBE samples with a diameter of 15 mm and length of 12 cm were prepared. The composition of all the samples was 44.5Pb-55.5Bi (wt%). After the samples were prepared and placed under constant temperature, four measurements of microstructure and mechanical properties at different time points were conducted to observe the variation rules. The selected time points were 3 days, 30 days, 90 days, and 180 days after sample preparation. Samples with four different cooling rates during sample preparation were divided into four groups: 0.1 K/min cooling rate for group 1 (G1), 1 K/min cooling rate for group 2 (G2), 5 K/min cooling rate for group 3 (G3), and 10 K/min cooling rate for group 4 (G4).

The phase composition of LBE was analyzed with XRD during the aging process of the LBE samples. An optical microscope (LV100ND/LV100NDA industrial metallographic microscope developed by Nikon, Japan) was used to observe the different phase proportions and morphological textures of LBE samples at different time points. The mechanical properties of aged LBE samples were measured using a compression tensile machine (SHIMADZU AGX-V 100 KN, Japan) at a compression rate of 10^−3^ s^−1^. Three samples were measured for each cooling condition. Each sample was cylindrical with a diameter of 5 mm and height of 5 mm. To investigate the rules of density variation of solidified LBE over time, density measurements of LBE were performed as well (Archimedes method was used). The surface of the sample was polished, and the oxide film was removed from the surface using ethanol and ultrasonic cleaning before measurement.

## 3. Results

### 3.1. Microstructural Characterization

**Figure 1:**
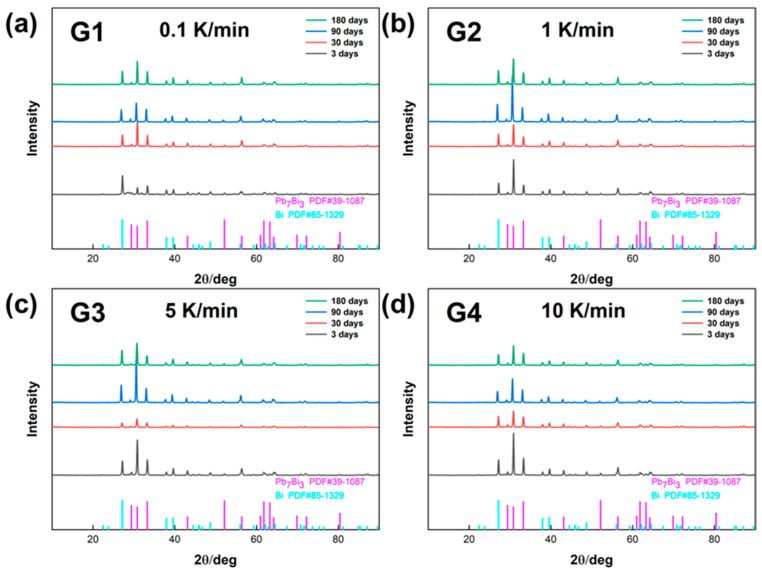
XRD patterns of the LBE samples obtained at different cooling rates. (**a**) 0.1 K/min LBE samples aging (0~180 d), (**b**) 1 K/min LBE samples aging (0~180 d), (**c**) 5 K/min LBE samples aging (0~180 d), (**d**) 10 K/min LBE samples aging (0~180 d).

The changes of the phase components of the LBE samples obtained at different cooling rates with aging time (0~180 days) were analyzed, and their XRD patterns are shown in Figure 1. From the four XRD patterns, it could be learned that the composition of the phases of LBE samples obtained at different cooling rates did not change significantly after LBE samples were subjected to a long period of quiescent aging, and their main components were still the LBE metal β-phase (Pb_7_Bi_3_) and γ-phase (Bi-rich phase).

The β-phase had a dense hexagonal crystal structure, which was characterized by high space utilization and coordination number, making the alloy structurally stable with a high density; the γ-phase had a rhombohedral–hexahedral crystal structure; and the γ-phase was a solid solution with a bismuth content of approximately 99.6% so that the density of the γ-phase was close to that of pure Bi.

**Figure 2:**
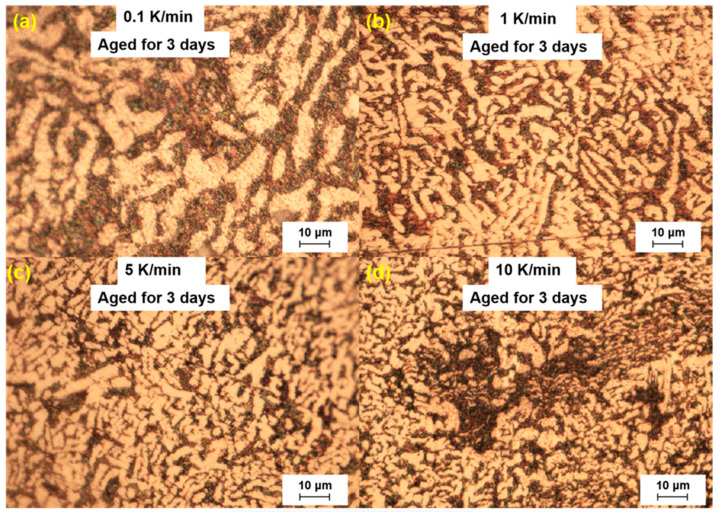
Microstructure of the LBE samples prepared with different cooling rates, aged for 3 days. (**a**) 0.1 K/min LBE samples aging 3 d, (**b**) 1 K/min LBE samples aging 3 d, (**c**) 5 K/min LBE samples aging 3 d, (**d**) 10 K/min LBE samples aging 3 d.

In the study, we analyzed the microstructure of the LBE samples prepared with different cooling rates. The analysis was performed three days after sample preparation. The microscopic results are shown in Figure 2. In the optical microscopy images, it was observed that the LBE samples obtained with different cooling rates all exhibited a microstructure with bright and dark areas. The bright areas mainly showed fishbone-like structures, considering that the γ-phase has better corrosion resistance than the β-phase. It can be confirmed that the bright phase was the γ-phase and the dark phase was the β-phase.

In the eutectic solidification process, the black β-phase precipitates first because of the low content of Bi in the β-phase. In its nucleation, the liquid phase is enriched with Bi, which creates conditions for the nucleation of the γ-phase. The γ-phase formed the nuclei in the β-phase, and then the β-phase and γ-phase connected to form the overall composition of the eutectic nucleus.

The eutectic alloy solidification process was nucleation → phase boundary equilibrium → short-range diffusion disrupts the equilibrium → growth → phase boundary equilibrium. This process was repeated until all solutions were transformed into a eutectic structure. As the temperature decreased, the secondary γ-phase precipitated from the pre-eutectic phase owing to the decreasing steady-state content of Bi in the β-phase. However, this process was slow and would be gradually completed during the long-term aging process after the cooling was completed.

In Figure 2, a large black β-phase could be seen from the 10 K/min sample, which indicated that atypical eutectic solidification occurs at very fast cooling rates, and this phenomenon led to an increase in the amount of Bi precipitated, which increased the final volume expansion.

In general, the cooling rate for the preparation of LBE samples affects the solidification process and grain size of metallic materials.

By comparing the microstructure of LBE under different cooling rates, it was found that with an increase in the cooling rate, the black β-phase gradually decreased, the fishbone structure increased, and the grain size of the eutectic phase became smaller. The main reason is mainly that when the cooling rate is large, LBE is more prone to nucleation during the solidification process, which made the size of the grains smaller, and this was more conducive to the precipitation of the secondary γ-phase, which also explained why the solidified LBE expansion was faster at a fast cooling rate.

The fishbone-like structures in the edge region of the images were fewer than those in the middle region because the areas closer to the edge experienced faster cooling rates and had more energy to nucleate and grow than the middle region. It could be concluded that the grain size was strongly dependent on the cooling rate, with lower cooling rates leading to larger grain sizes and vice versa. Meanwhile, an increase in the cooling rate enhanced the conversion of β-phase to γ-phase and the precipitation of the γ-phase. The volume expansion rate of the LBE was also enhanced. Considering that the density of the β-phase was 11.17 g/cm^3^, and the density of the γ-phase was 9.75 g/cm^3^, the γ-phase precipitation required more space, resulting in an expansion of LBE volume, which is also mentioned in other literature [13].

**Figure 3:**
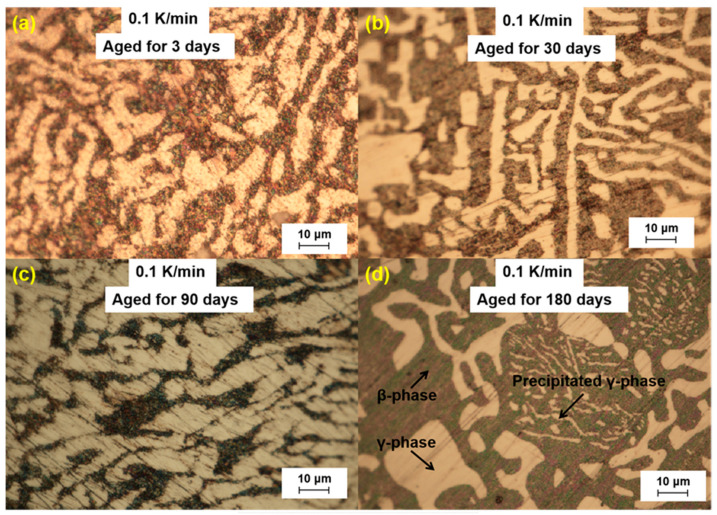
Optical microscope images of microstructure evolution after different aging times for the LBE samples at a cooling rate of 0.1 K/min. (**a**) 0.1 K/min LBE samples aging 3 d, (**b**) 0.1 K/min LBE samples aging 30 d, (**c**) 0.1 K/min LBE samples aging 90 d, (**d**) 0.1 K/min LBE samples aging 180 d.

**Figure 4:**
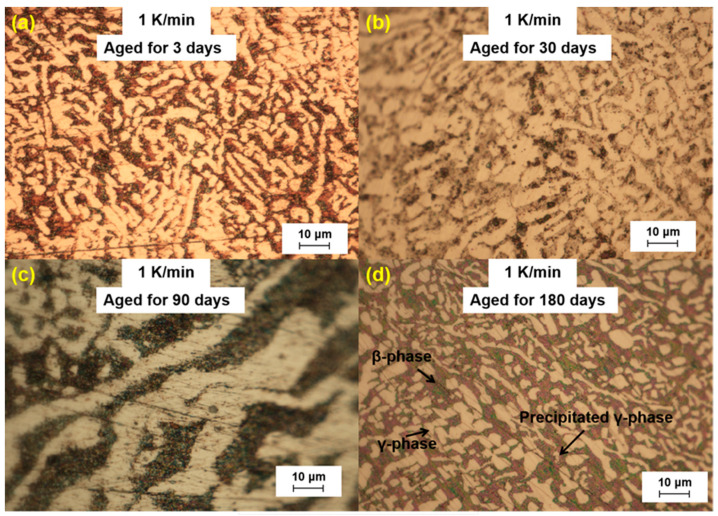
Optical microscope images of microstructure evolution after different aging times for the LBE samples at a cooling rate of 1 K/min. (**a**) 1 K/min LBE samples aging 3 d, (**b**) 1 K/min LBE samples aging 30 d, (**c**) 1 K/min LBE samples aging 90 d, (**d**) 1 K/min LBE samples aging 180 d.

**Figure 5:**
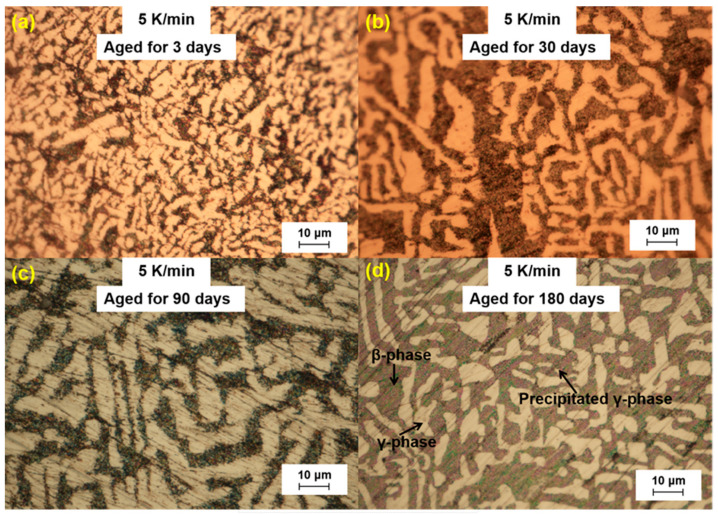
Optical microscope images of microstructure evolution after different aging times for the LBE samples at a cooling rate of 5 K/min. (**a**) 5 K/min LBE samples aging 3 d, (**b**) 5 K/min LBE samples aging 30 d, (**c**) 5 K/min LBE samples aging 90 d, (**d**) 5 K/min LBE samples aging 180 d.

**Figure 6:**
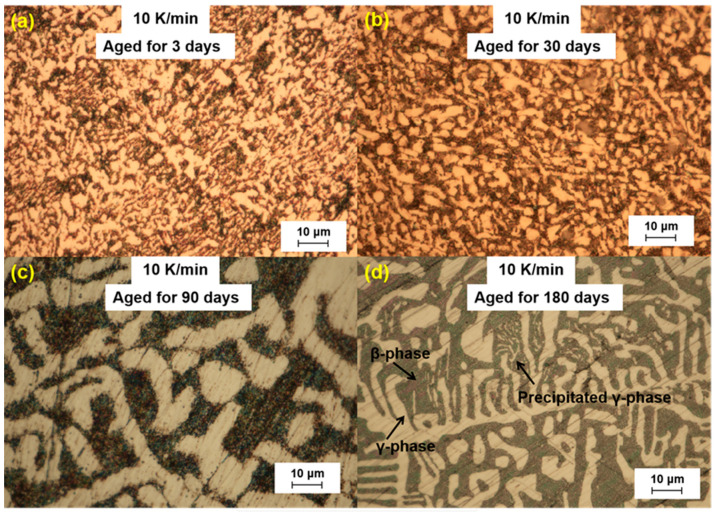
Optical microscope images of microstructure evolution after different aging times for the LBE samples at a cooling rate of 10 K/min. (**a**) 10 K/min LBE samples aging 3 d, (**b**) 10 K/min LBE samples aging 30 d, (**c**) 10 K/min LBE samples aging 90 d, (**d**) 10 K/min LBE samples aging 180 d.

We further analyzed the microstructural changes of the LBE samples with aging time (0~180 d) at different cooling rates. The optical microscope images of the LBE samples after aging at different cooling rates are shown in Figure 3, Figure 4, Figure 5 and Figure 6. As can be seen from the figures, the microstructure of LBE samples prepared with four different cooling rates did not change significantly after 90 d of static aging. When the aging time was extended to 180 d, a distinct white γ-phase was observed in the large black β-phase, confirming the phase-transition mechanism. With the extension of aging time, more holes appeared on the surface of the samples, caused by the volume expansion of the LBE samples during the long-term aging process. The formation of holes on the surface of the samples indicated that LBE underwent volume expansion during long-term aging.

### 3.2. Mechanical Properties

**Figure 7:**
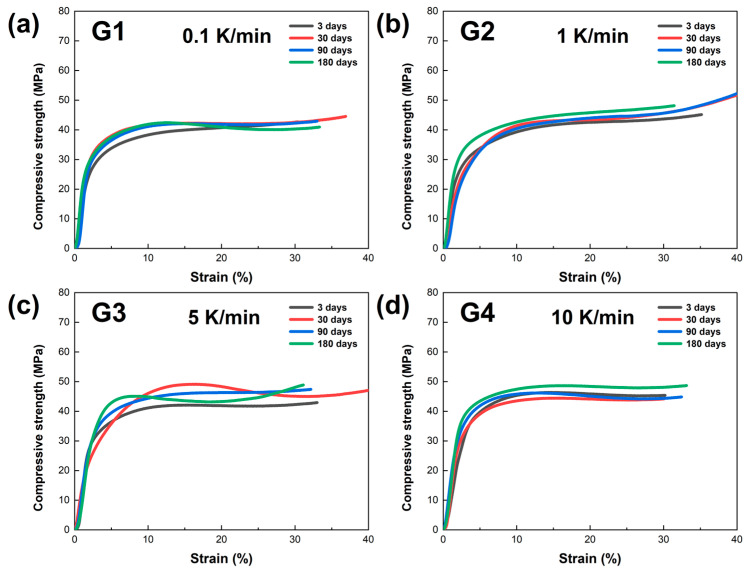
Mechanical compression curves of the LBE samples obtained at different cooling rates. (**a**) 0.1 K/min LBE samples aging (0~180 d), (**b**) 1 K/min LBE samples aging (0~180 d), (**c**) 5 K/min LBE samples aging (0~180 d), (**d**) 10 K/min LBE samples aging (0~180 d).

In this study, the mechanical properties of LBE samples obtained at different cooling rates were evaluated through compression testing. Mechanical compression curves of the LBE samples at various aging times (0~180 days) were obtained, and the results are presented in Figure 7. As shown in Figure 7a, the compressive strength of the LBE samples cooled at 0.1 K/min exhibits linear growth in the initial period, indicating that the material is in the elastic deformation stage during this period. As the strain increases, the curves gradually deviate from linearity and enter the yield stage. At this point, the material begins to undergo plastic deformation. The curves of different colors in the figure represent the test results at different times (3, 30, 90, and 180 days). As shown in Figure 7b–d, with an increase in the cooling rate, the compressive curves of the LBE samples exhibited similar growth trajectories. However, it is noteworthy that as the cooling rate increased, the compressive strength of LBE showed an upward trend.

The comparison of the four images indicates that the compressive strength of the LBE samples obtained at the same cooling rates does not change significantly with increasing aging time. In addition, as the cooling rate increases, the maximum compressive strength of the LBE samples generally shows an upward trend. This is because the increase in the cooling rate greatly promotes the enhancement of the nucleation rate and a reduction in the grain growth time, resulting in significant refinement of the grain size of the LBE after cooling. Since the yield strength of a material is inversely proportional to the grain size, the compressive strength of the LBE samples exhibits an upward trend with the increasing cooling rate.

Through mechanical compression experiments on LBE samples under different cooling rates, we obtained valuable experimental data. The experimental results indicate a significant correlation between the cooling rate and yield strength of the LBE: the faster the cooling rate, the greater the yield strength of the LBE. The yield strength is the stress value at which a material begins to undergo significant plastic deformation when subjected to force, and it reflects the material’s ability to resist deformation. In the operating environment of a reactor with LBE as a coolant, its changes of yield strength can have a significant impact on the structural safety and stable operation of the reactor. Therefore, based on the aforementioned experimental results and safety considerations, we recommend that the cooling rate of the reactor should not be too fast.

**Figure 8:**
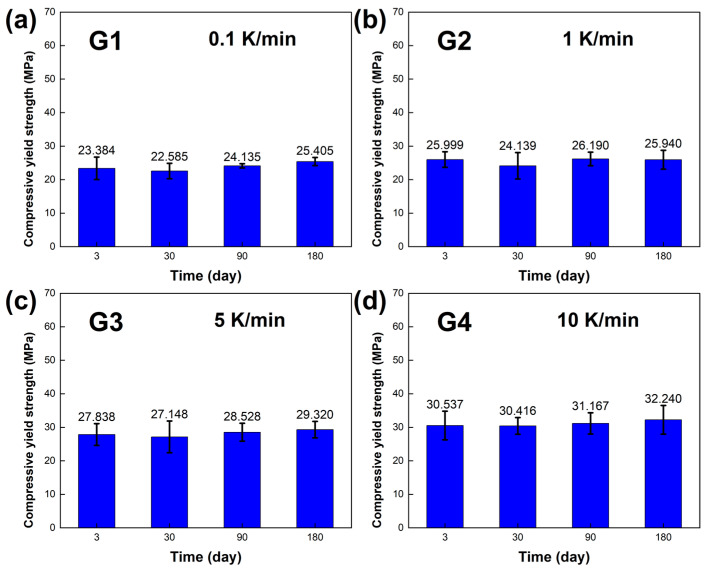
Compressive yield strength σ of the LBE samples obtained at different cooling rates. (**a**) 0.1 K/min LBE samples aging (0~180 d), (**b**) 1 K/min LBE samples aging (0~180 d), (**c**) 5 K/min LBE samples aging (0~180 d), (**d**) 10 K/min LBE samples aging (0~180 d).

**Figure 9:**
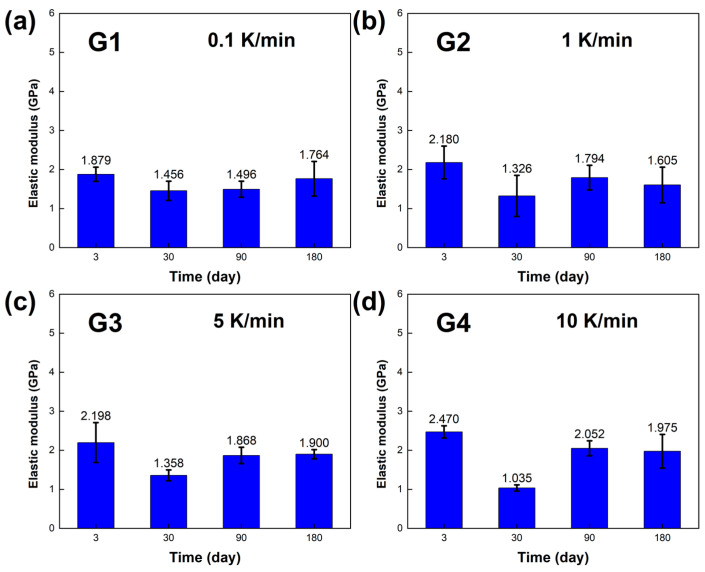
Elastic modulus E of the LBE samples obtained at different cooling rates. (**a**) 0.1 K/min LBE samples aging (0~180 d), (**b**) 1 K/min LBE samples aging (0~180 d), (**c**) 5 K/min LBE samples aging (0~180 d), (**d**) 10 K/min LBE samples aging (0~180 d).

The mechanical properties, including the compressive yield strength σ and elastic modulus E of the LBE, were obtained, and the results are presented in Figure 8 and Figure 9. At room temperature and pressure, the elastic modulus E of the LBE samples obtained at different cooling rates ranged from 1~3 GPa, and the compressive yield strength σ of the LBE samples ranged from 22~33 MPa at a compression rate of 10^−3^s^−1^.During the aging process of up to 180 days, Figure 8a and Figure 9a showed that the compressive yield strength σ and elastic modulus E of the sample had little change when the cooling rate was 0.1 K /min. The compressive yield strength σ of the long-term aging sample showed little change compared with that of the sample aged within three days. The average compressive yield strength σ was 23.877 MPa, and the average elastic modulus E was 1.649 GPa. Figure 8b and Figure 9b show the compressive yield strength σ and elastic modulus E, respectively, at a cooling rate of 1 K/min. The overall results did not show significant changes. The average compressive yield strength σ was 26.150 MPa, and the average elastic modulus E was 1.726 GPa. Figure 8c and Figure 9c show the compressive yield strength σ and elastic modulus E at a cooling rate of 5 K/min. The average compressive yield strength σ was 28.209 MPa, and the average elastic modulus E was 1.831 GPa. Figure 8d and Figure 9d show the compressive yield strength σ and elastic modulus E at a cooling rate of 10 K/min. The average compressive yield strength σ was 31.090 MPa, and the average elastic modulus E was 1.883 GPa. As the aging time increased, there was no significant difference in the compressive yield strength σ of the LBE samples with the same cooling rate, and only a certain degree of fluctuation. The elastic modulus also shows similar results, indicating that aging time has a minor effect on the compressive yield strength σ and elastic modulus E of LBE. With an increase in cooling rate, the compressive yield strength σ shows an upward trend, while the elastic modulus is not significantly affected, with a small amplitude of fluctuation. It should be noted that the samples in the experiment are affected by various factors, such as storage temperature, handling, and instrumental errors. Therefore, it is inevitable that there will be slight uncertainties in the measurements.

**Figure 10:**
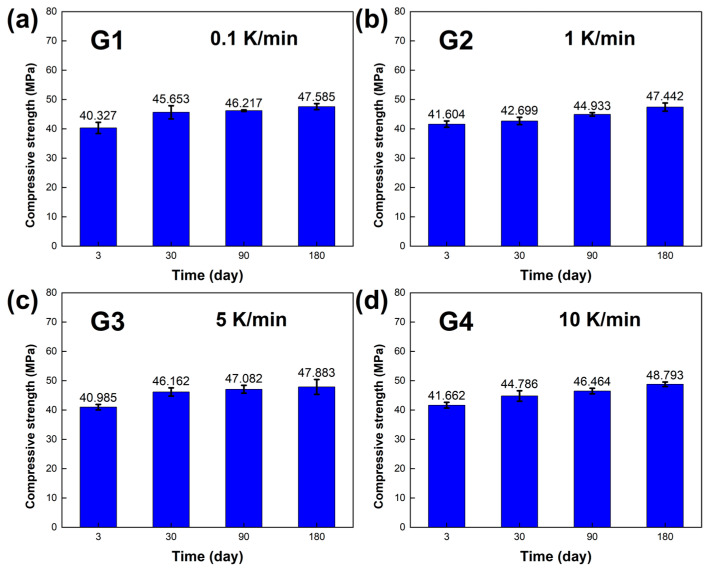
Compressive strength of the LBE samples obtained at different cooling rates. (**a**) 0.1 K/min LBE samples aging (0~180 d), (**b**) 1 K/min LBE samples aging (0~180 d), (**c**) 5 K/min LBE samples aging (0~180 d), (**d**) 10 K/min LBE samples aging (0~180 d).

**Figure 11:**
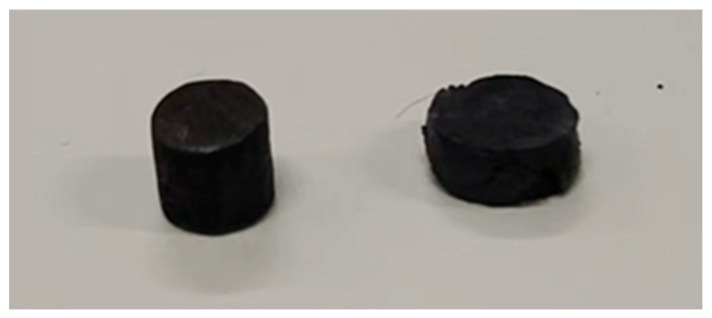
A sample before testing and a sample after 3 mm compression.

The compressive strength of the LBE was also obtained in the experiments, and the results are shown in Figure 10. At normal temperature and pressure, it could be seen that after long-term aging, the compressive strength of the LBE samples improved significantly, and the differences in compressive strength between the LBE samples at different cooling rates decreased, which indicated that the hardness of the LBE samples increased. Figure 11 illustrates the sample before the test and after a compression of 3 mm.

### 3.3. Density Results

**Figure 12:**
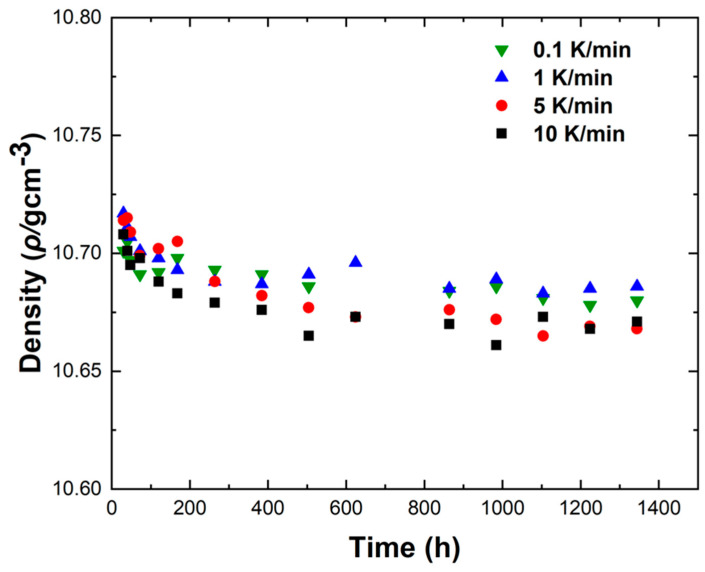
Measurement of density changes of LBE samples obtained at different cooling rates.

Figure 12 shows the density variations of LBE samples obtained with different cooling rates. The first measurement time was 30 h after the sample preparation, and the last measurement time was 1344 h after the sample preparation. It can be seen from Figure 12 that the densities measured for the first time were around 10.72 g/cm^3^, the densities of the samples showed a gradual decline as the aging time increased, and the densities measured for the last time were around 10.68 g/cm^3^. The densities of all samples gradually became stable after 1344 h of aging. After long-term aging, the decrease in density led to the volume expansion of LBE during the aging process after solidification. The density changes of the samples obtained with rapid cooling rates (5 K/min and 10 K/min) after long-term aging were significantly greater than those of slow cooling rates (0.1 K/min and 1 K/min). Since atypical eutectic solidification would occur at very fast cooling rates, this phenomenon led to an increase in the amount of Bi precipitation, resulting in greater volume expansion and larger density decrease.

**Figure 13:**
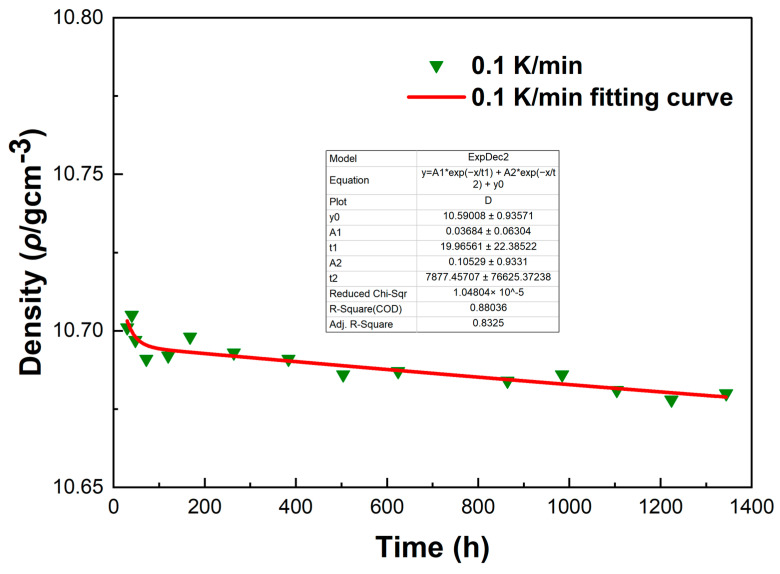
The fitting curve of density variation with time for the LBE sample at a cooling rate of 0.1 K/min.

**Figure 14:**
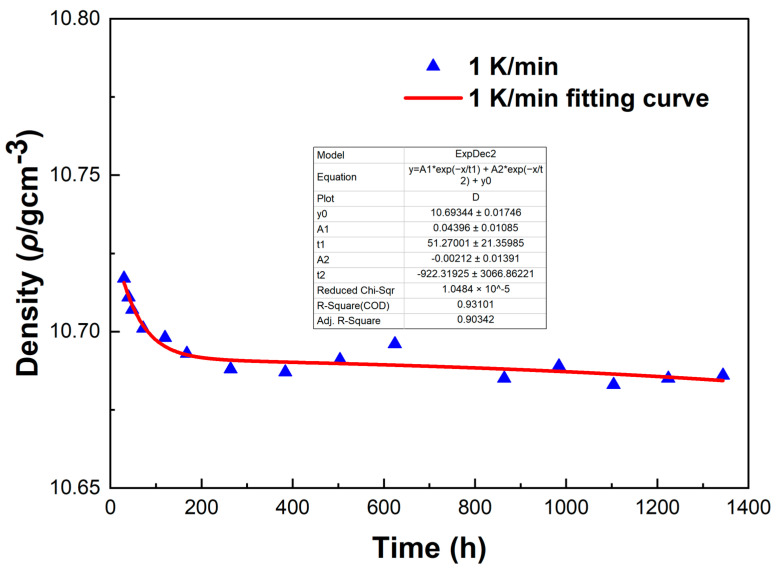
The fitting curve of density variation with time for the LBE sample at a cooling rate of 1 K/min.

**Figure 15:**
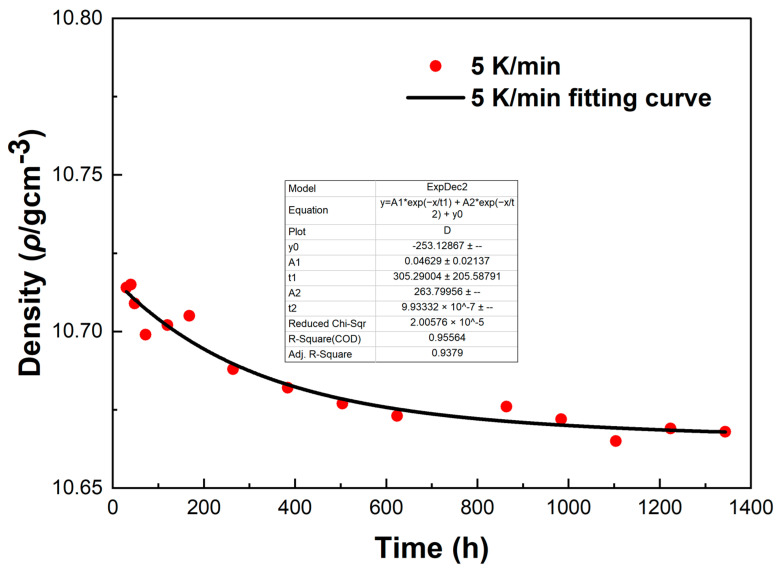
The fitting curve of density variation with time for the LBE sample at a cooling rate of 5 K/min.

**Figure 16:**
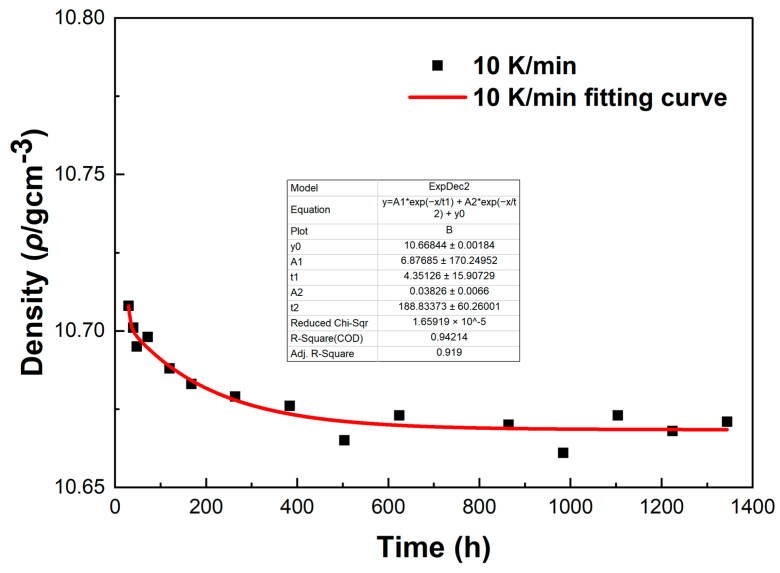
The fitting curve of density variation with time for the LBE sample at a cooling rate of 10 K/min.

To investigate the errors in the density measurement, we conducted a fitting curve analysis of the density data points of the LBE at different cooling rates. The resulting fitting curves, which illustrate the variation of density with time, are shown in Figure 13, Figure 14, Figure 15 and Figure 16.

From Figure 13, Figure 14, Figure 15 and Figure 16, it can be observed that these fitting curves effectively demonstrate the trend of the LBE density variation with time under different cooling rates. However, in the density fitting curves, we noticed that individual data points exhibited anomalies. These anomalous data points may be attributed to occasional errors during the experiment, the influence of the measurement equipment, or local characteristic differences within the sample. Moreover, we can also determine the errors in the density measurement from the fitting curve figures, and these errors are all within an acceptable range. In summary, we believe that these density data points have good accuracy and credibility.

## 4. Discussion

The lead-bismuth cooled fast reactor is a fourth-generation advanced nuclear energy system that utilizes lead-bismuth eutectic (LBE) as the coolant. Compared with traditional coolants, LBE exhibits numerous advantages, such as excellent neutronic properties, low melting point, high boiling point, high thermal conductivity, and strong chemical inertness. However, LBE undergoes volume expansion during the aging process after solidification. The volume expansion effect of LBE can cause damage to the components and containers within the reactor. Therefore, an in-depth exploration of the expansion mechanism of LBE is crucial for a comprehensive understanding of its properties. Due to the continuous variation in the expansion rate of LBE in practical engineering experiments, there is significant uncertainty in its key physical properties, such as yield strength, elastic modulus, and density. Moreover, reliable experimental data for reference are lacking.

Previously, although some scholars have explored the aging behavior mechanism of LBE, these studies have certain limitations. Specifically, the selected aging periods were relatively short and did not cover long spans, such as 180 d. A short aging period can only reflect the property changes of the material in the initial stage, making it difficult to reveal the evolution law of the microstructure and variation trend of the mechanical properties during the long-term aging process. Compared to previous studies, our team’s research is unique. In this context, we carried out a 180-day study on the topic of “the effect of aging on the microstructure and mechanical properties during the aging process after LBE solidification”. We systematically investigated the changes in the microstructure and mechanical properties of LBE under different operating conditions, i.e., different combinations of cooling rates and aging times. We obtained microstructural images and physical property parameters (yield strength, elastic modulus, density, and compressive strength) of the LBE aging process (3~180 d) after solidification. Our experimental results enrich the research of the aging behavior mechanism of LBE during long-term aging periods.

## 5. Conclusions

In the study, LBE samples were prepared with different cooling rates and treated with static aging. The microstructures, compressive mechanical properties, and density change of LBE after solidification were investigated systematically and quantitatively, and the main conclusions were as follows:

(1) The phase structure of LBE samples obtained with different cooling rates after aging was mainly the β-phase (Pb_7_Bi_3_) and γ-phase (Bi-rich phase).
(2) The volume expansion of LBE after solidification during long-term aging was due to the transition from β-phase to a stable γ-phase. After aging for 180 days, it was found that the white γ-phase precipitated from the black β-phase, which further confirmed the phase transition mechanism. With the increase in cooling rate for the preparation of samples, the precipitation of the γ-phase increased, and the expansion was enhanced. Additionally, the volume expansion of LBE increased with the increase in aging time.
(3) As the aging time increased, there was no significant difference in the compressive yield strength σ of the LBE samples with the same cooling rate and only a certain degree of fluctuation. The elastic modulus E also shows similar results, indicating that aging time has a minor effect on the compressive yield strength σ and elastic modulus E of the LBE. With the increase of cooling rate, the compressive yield strength σ shows an upward trend, while the elastic modulus E is not significantly affected, with a small amplitude of fluctuation.
(4) During the aging process after solidification, the overall density of the LBE samples exhibits a decreasing trend. The ranges of density fluctuations under fast cooling rates (5 K/min and 10 K/min) are significantly larger than those under slow cooling rates (0.1 K/min and 1 K/min). The decrease in density leads to volume expansion of LBE during the aging process after solidification, and an increase in the cooling rate exacerbates the volume expansion of LBE.
(5) Based on the mechanical compression experiments conducted on lead-bismuth eutectic alloy (LBE) samples at different cooling rates, we found that the faster the cooling rate, the greater the yield strength of LBE after solidification. For safety reasons, it is recommended that the cooling rate of the reactor should not be too fast.

## Data Availability

Data are available on request from the corresponding author, Xi Huang.
